# Clinical Network Systems Biology: Traversing the Cancer Multiverse

**DOI:** 10.3390/jcm12134535

**Published:** 2023-07-07

**Authors:** Isa Mambetsariev, Jeremy Fricke, Stephen B. Gruber, Tingting Tan, Razmig Babikian, Pauline Kim, Priya Vishnubhotla, Jianjun Chen, Prakash Kulkarni, Ravi Salgia

**Affiliations:** 1 Department of Medical Oncology and Therapeutic Research, City of Hope National Medical Center, Duarte, CA 91010, USA; 2 Department of Pharmacy, City of Hope National Medical Center, Duarte, CA 91010, USA; 3 Department of Medical Oncology, City of Hope Atlanta, Newnan, GA 30265, USA; 4 Department of Systems Biology, City of Hope National Medical Center, Duarte, CA 91010, USA

**Keywords:** team medicine, precision medicine, cancer systems biology, clinical network systems biology

## Abstract

In recent decades, cancer biology and medicine have ushered in a new age of precision medicine through high-throughput approaches that led to the development of novel targeted therapies and immunotherapies for different cancers. The availability of multifaceted high-throughput omics data has revealed that cancer, beyond its genomic heterogeneity, is a complex system of microenvironments, sub-clonal tumor populations, and a variety of other cell types that impinge on the genetic and non-genetic mechanisms underlying the disease. Thus, a systems approach to cancer biology has become instrumental in identifying the key components of tumor initiation, progression, and the eventual emergence of drug resistance. Through the union of clinical medicine and basic sciences, there has been a revolution in the development and approval of cancer therapeutic drug options including tyrosine kinase inhibitors, antibody–drug conjugates, and immunotherapy. This ‘Team Medicine’ approach within the cancer systems biology framework can be further improved upon through the development of high-throughput clinical trial models that utilize machine learning models, rapid sample processing to grow patient tumor cell cultures, test multiple therapeutic options and assign appropriate therapy to individual patients quickly and efficiently. The integration of systems biology into the clinical network would allow for rapid advances in personalized medicine that are often hindered by a lack of drug development and drug testing.

## 1. Introduction

Cancer is a complex disease that is caused by a dysfunction of normal cell biology through genetic and non-genetic changes including epigenetic changes that corrode the cell’s ability to promote cell death, resulting in a process of dysregulated growth and proliferation. Every year, approximately over 1.9 million people are diagnosed with cancer and 609,820 die from cancer in the United States alone [1]. The discovery of new diagnostic tools, immunotherapy, and novel therapies has helped to reduce the cancer death rate by 33% since 1991, but despite this positive milestone, the improvement in outcomes has not been uniform across all tumor types [1]. This is largely in part due to the heterogeneity of cancer as a multi-modal disease that is driven by a collection of genetic and non-genetic mechanisms, which means tumors from a single tissue type do not respond to the same therapies despite similar histological profiles [2,3,4]. Therefore, considerable effort has been invested over the last 20 years to understand the biology of cancer and more importantly cancer within individual patients to decipher the heterogeneity of cancer types [3,5,6,7]. The revolution in next-generation sequencing, liquid biopsy, single-cell sequencing, proteomics, and other novel diagnostic techniques has generated large libraries of whole genome, transcriptome, epigenetic, proteomic, and metabolomic data [3,5,6,7,8]. However, the relationship between the individual gene and protein discoveries is not intrinsic in affecting tumor pathology, and often times, intricate cascade effects in transcriptional, translational and post-translational modification limit therapeutic efficacy [9,10]. In essence, effective cancer therapeutics cannot be achieved through understanding a cancer’s individual parts but requires a systems biology approach where large cross-collaborations of multi-modal scientists, clinicians, and experts collaborate to understand the entirety of the oncogenic network.

Systems biology at its foundation is comprehending that the whole is greater than the sum of its individual parts and is a heuristic process of collaboration, prediction, and discovery that has yielded several scientific discoveries in the last century [11,12]. Within a biological system, key processes are necessary for system-level insight and understanding including system structures, systems dynamics, the control method, and the design method as initially described by Kitano et al. [13]. Within the cancer systems biology paradigm, the system structures can be separated into five networks including the gene regulatory network, molecular network, cellular network, organ network, and clinical and research network. The systems dynamics process aims to understand how cancer as a complex system of abnormal cell growth behaves and changes over time from an initial set of conditions [14]. The cancer control method of systems biology relies on modulating the state of the cell to limit cancer growth or induce apoptosis to validate potential therapeutic options [15,16]. The highest level of cancer systems biology is a design method or design principles where multi-dimensional models, from in silico mathematical models to cell cultures to organoids to mouse PDXs, are constructed to mimic and mirror the oncogenic properties of individual patients or a cohort of patients so that therapies can be tested and applied based on the definitive initial conditions of the tumor [16,17,18]. Due to this multi-scale and multi-modal persistence of cancer, we propose a novel highly adaptive approach of clinical network cancer systems biology that integrates basic science expertise and novel methodology with physician-level expertise and patient access to achieve the dream of personalized medicine. With the advent of modern technology, especially machine learning and artificial intelligence (AI), it is noticeably clear that cancer systems biology ought to take on an integrated approach where preclinical biology, patient translational specimens, and clinical care are all merged under a singular umbrella.

## 2. Systems Biology in Cancer

One of the primary challenges in cancer is that it cannot be understood through a simplistic lens due to the nonlinear nature of the disease process and its subsequent evolution. At the organ level, cancers exhibit differential patterns, and more evidence has shown that cancer metastasis may have a deterministic pattern to its chaotic process where certain genotypes show a preference toward target organs [19]. Furthermore, the tumor tissue and its tumor microenvironment (TME) vary by cancer type, and recent evidence shows that the TME may have an active role in the proliferation, migration, invasion, survival, angiogenesis, and EMT within the cancer cell network [20]. This is further complicated by protein signaling networks and biochemical signaling pathways involved in cancer progression that are difficult to predict and overcome therapeutically due to distinct perturbations in genotypes and phenotypes that drive their formation and interaction [21,22]. At the lower magnification, genomic instability in DNA repair and maintenance mechanisms as well as the disruption of epigenetic regulators has led to the discovery of several genomic alterations and chromatin modifications. This has unfortunately led to a high failure rate with only 6.7% of therapies reaching the phase II trial phase with regulatory approval between 2009 and 2018 [23]. Ultimately, the issue of cancer drug discovery is two-fold in that while with the help of next-generation sequencing, large cohorts of patients have been identified with novel targeted therapeutic options such as NSCLC EGFR-mutated patients or BRCA-2 positive breast cancer, there were also numerous cohorts of patients discovered with genomic alterations that have no clinically proven drug options such as TP53, ARID1A or PIK3CA [24].

**Figure 1:**
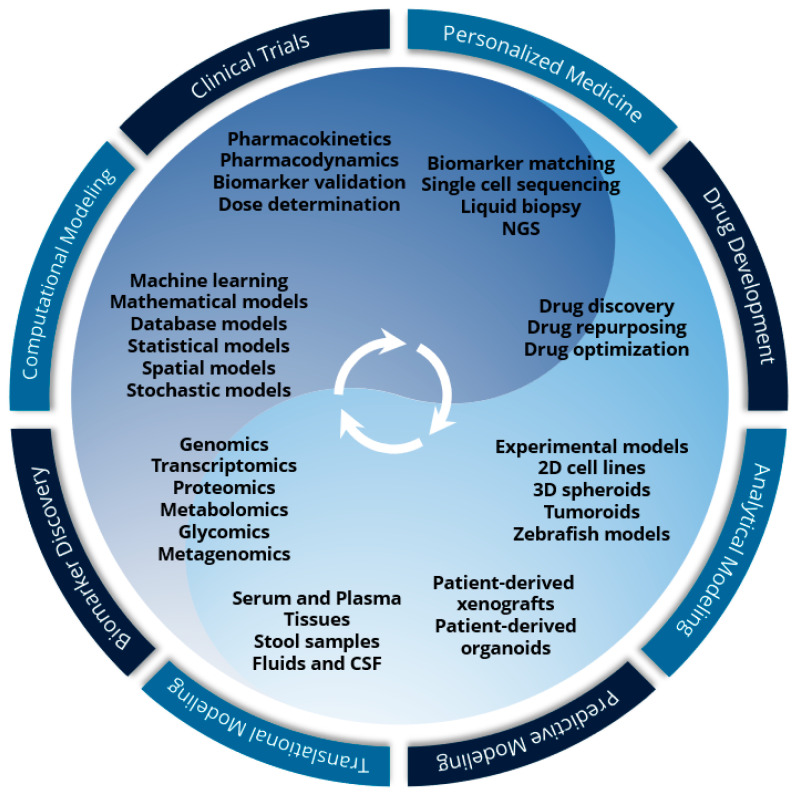
The life cycle of cancer systems biology drug discovery.

The discovery of novel therapeutics based on recent preclinical biological discoveries is an iterative process within cancer systems biology that can be represented as a life cycle of research that combines wet-lab and dry-lab efforts to arrive at validated therapeutics (Figure 1). While traditionally systems biology begins its life cycle at preclinical basic research, this is different in cancer in that there is a wide berth of data that is publicly available from large cancer databases such as TCGA and publicly available results from individual large cohort studies. This makes the life cycle of cancer systems biology more fluid in that initial discoveries or drug targets can be made prior to any wet-lab experiments through bioinformatics analyses and in silico modeling. Nevertheless, wet-lab analytical modeling involving cell lines, 3D spheroids, tumoroids, and in vivo experiments is a required stepping stone toward verifying an underlying therapeutic hypothesis regardless of whether the foundation of that hypothesis was based on previous preclinical or clinical knowledge. Subsequently, predictive modeling and translational research go hand in hand in validating the clinical efficacy and viability of any therapeutic approach. This is then followed by biomarker discovery and computational modeling where potential therapeutics attempt to find the “best-fit niche” for their mechanism of action. However, it is important to underscore that the cancer systems biology life cycle is nonlinear, and each step may flow back into the previous step where further analytical modeling and predictive modeling work is required based on the computational and biomarker findings, which in turn may require new hypotheses to be made. This has further importance in clinical trials and personalized medicine where initial findings of the therapeutic in a clinical population such as toxicity or various omics profiling may yield results that require further drug optimization or drug repurposing.

The arrival of next-generation sequencing in the clinical setting has allowed for the further stratification of individual cancer types beyond their histology or tumor locale. However, as mentioned previously, cancer systems biology is complicated by the fact that individual components of data do not represent the entire network of the cancer system. While genomic data has been valuable in developing targeted therapies and stratifying patients by biomarkers, it is not uniform with actionable mutation rates in patients varying from 10.8% to 90.6% depending on cancer type [25]. This leaves large cohorts of patients without viable therapeutic options. A recent example is EGFR to SCLC transformation following osimertinib therapy, which underscores the importance of non-genetic mechanisms at play in cancer resistance [26]. The underlying challenge for this beyond identifying the possible drug candidates and novel therapeutic approaches is clinical trial cost and a lack of clinical trial integration into the oncology standard of care, which in turn further increases clinical trial costs [27,28,29]. This is in part due to the traditional clinical trial model where cohorts of patients at different sites especially in the community network are screened for individual trials separately to identify an individual with a biomarker that is possibly present in less than 1% of that cancer population [27,28,29]. The implementation of large umbrella trials such as the Lung-MAP, ALCHEMIST, or NCI-MATCH trials that aim to screen patients’ biomarkers and match the patients to appropriate therapies have been successful in the academic setting [30]. However, it has been reported that 40% of patients were more than 60 min away from a clinical trial location, which is a central issue in increasing NCI-MATCH trial recruitment in the community network setting [31,32]. This is further complicated by the lack of access to the community practice patients from the trial and drug development perspective in that often, the complex community network patients do not have access to trials that address their biomarker [33].

**Figure 2:**
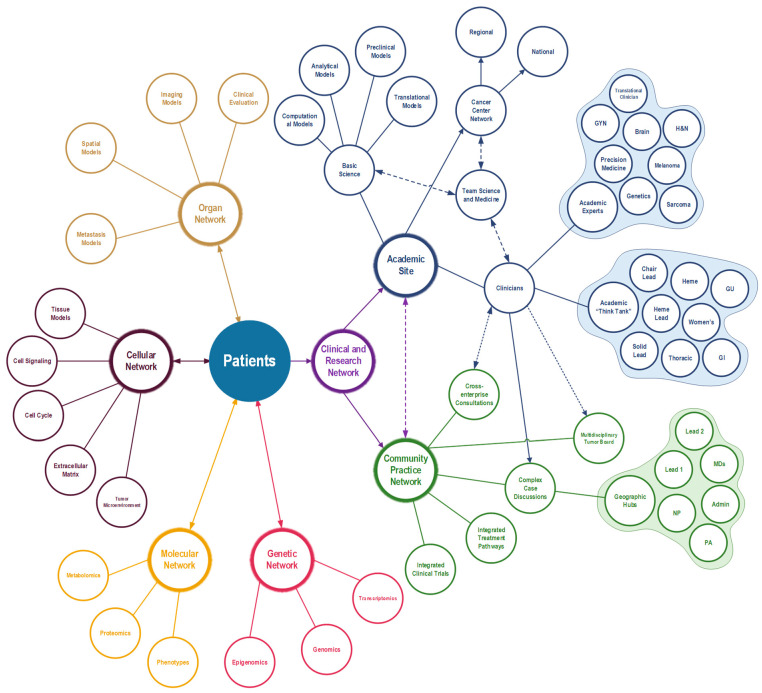
Clinical Network Systems Biology framework that integrates the biological networks with the clinical and research networks.

We believe the solution to these issues is a novel approach that integrates cancer systems biology with a concept that we previously identified called “Team Medicine” [34,35]. Team Medicine is a cross-collaborative effort to integrate basic scientists with clinicians to drive forward rapid-pace translational research. The merging of Team Medicine and cancer systems biology would result in a new paradigm called Clinical Network Systems Biology where the academic site, the clinical community network, and basic scientists at a research center would integrate under one umbrella to discover, develop, and test novel therapeutics at a rapid pace to achieve more personalized medicine (Figure 2). The framework embodies the four biological networks involved in cancer including the organ network, cellular network, molecular network, and gene regulatory network, and it combines it with the clinical and research network that encompasses the primary academic site and community practice network.

In the subsequent sections, we will delve deeper into the two components that comprise this framework by looking at the individual parts of the biological network that drive the patients’ cancer and the various strategies that can be utilized in the clinical network to enhance the basics of systems biology toward precision medicine.

### 2.1. Biological Network in Cancer Systems Biology

Clinical Network Systems Biology is analogous to the Matryoshka nesting dolls: a set of wooden dolls of increasing size placed one inside another. Thus, Matryoshka serves as a great metaphor for a complex system. Analytically speaking, the metaphor is especially well suited since it is likened to thinking in systems. Relatively speaking, a system may be defined as an interconnected set of components that are organized toward a specific function or purpose. Complex systems are systems within a system. Indeed, a Clinical Network may be thought of as a complex system itself. Here, the biological network may be perceived as comprising the inner (smallest) doll representing a single cell with its gene network, i.e., the gene regulatory network (GRN) together with the non-genetic, protein interaction network (PIN), which is followed by the next (bigger) doll representing the cellular networks to form tissues that comprise the individual organs and, finally, a bigger doll representing a network of organs that constitute an individual. Thus, it follows that a Clinical Network is a complex system comprising many systems which may interact with each other with dependencies, competitions, relationships, or other types of interactions such as feedback loops between their parts or between the system and its environment. These interactive systems are traditionally called complex adaptive systems (CAS) where the biological behavior of one component does not predict the behavior of the other components. CASs are capable of self-organization that adapts to their environmental stimuli, which increases their chances of survival. Therefore, due to the unpredictable and temporal nature of these systems, they cannot be studied with traditional tools and require analysis using nonlinear dynamical models that can accurately predict emergent behaviors, cellular plasticity, and heterogeneous cells.

GRN (Gene Regulatory Network): At the principal level, a GRN is a group of genes that are characterized by gene expression and linked to one another through target gene nodes that regulate a specific cell function. Such interactions are genetically “wired” to ensure transgenerational transfer with high fidelity. Regulators of gene expression include transcription factors (TFs) that typically bind specific DNA sequence motifs and transcriptional regulators that typically interact with the basic transcriptional machinery and specific transcription factors. Both TFs and regulators can act as either activators of gene expression or as repressors that repress gene expression. Other molecules that may also play important roles in regulating gene expression include RNA-binding proteins and regulatory RNAs. Elucidating the intricate regulatory relationships between TFs, transcriptional regulators and their targets is essential to understand cellular functions such as cell growth and division, differentiation, and development. They can also help shed light on evolution, especially in the past half a billion years or so [36]. Furthermore, identifying GRNs can also aid in understanding how the dysregulation of gene expression contributes to complex heritable diseases as well as diseases such as cancer that have both genetic and non-genetic underpinnings [37,38].

PIN (Protein Interacting Network): The proteins that result from differential gene expression regulated by the GRNs interact with their cognate partners to form cellular PINs. While it was initially believed that PIN configurations occur randomly, Barabási and colleagues showed that PINs have a “scale-free” architecture in which the degree distribution P(k) expresses a power-law behavior as a function of the degree k [39,40]. A major advantage of scale-free networks is that they are largely resistant to random node failure, but they are vulnerable to critical hub failures [39].

Intrinsically disordered proteins (IDPs) are proteins that lack unique 3D structures and constitute a significant fraction of the proteome [41,42]. Because IDPs exist as conformational ensembles (are highly malleable), they can interact with multiple partners [43]. Consistent with their unique ability to interact with multiple partners, IDPs occupy hub positions in the scale-free network and play critical biological roles including transcriptional regulation [44,45,46]. Furthermore, they also regulate several key processes such as cell cycle regulation and facilitate phenotypic plasticity [47,48,49,50]. Nevertheless, IDP dysregulation of expression can often bring about non-specific interactions and generate phenotypic plasticity due to PIN modulation. This heuristic can often discover dormant pathways in the network and result in phenotypic variability. When the environmental stressors are removed, the IDPs are capable of reconfiguring the PIN to its original state, which suggests a non-genetic mechanism in phenotypic reversal. However, when the stressors persist, they can result in chronic network frustration through the acquisition of DNA mutations and other genetic alterations, which can result in permanent phenotypic alterations. This pinpoints the genetic/non-genetic duality in nature such as the evolution of drug resistance in tumor cells. This duality helps us understand how non-genetic mechanisms are involved in acquired resistance through irreversible genetic alterations at the single cell level. 

Cellular Network: Individual cells, both in normal healthy tissue as well as in diseased tissue such as cancer for example, do not exist as individuals: they live in communities with other cells be it in their natural tissue environment or the tumor microenvironment. Therefore, they exhibit group behavior which can significantly influence their fitness. Thus, it is imperative to gain a systems perspective to fully understand their group behavior, leading to the expected physiological output or how cancer cells exploit group behavior to evade the toxic effects of a drug to eventually develop drug resistance. Nonetheless, previous studies have not investigated drug resistance from such a systems-level perspective. Most studies employ a reductionist approach focusing on a gene target, its mutated version(s), a pathway, or a small molecule. Alternatively, they endeavor to develop “intermittent/adaptive” therapy by studying group behavior at the population level but do not consider the role of individual molecules or the associated pathways.

Organ network: The human body is a complex interconnected organ system where individual organs have their own morphology and functional diversity, which leads to temporary, shifting, nonlinear output biological changes. This process is interlinked in that one organ in the system has a direct effect on the behavior of the other systems. The multi-component organ systems regularly interface with one another through continuous feedback mechanisms and throughout varying scales of space and time to arrive at a precise physiological output. The lack of such coordinated interactions and communications can lead to the malfunction of individual systems or the entire organism [51]. Thus, it follows that a systems perspective rather than a reductionist approach is required to gain an in-depth understanding of the integrated physiologic function, which is an emergent phenomenon resulting from interactions between the diverse organ systems. Indeed, in recent years, a new field called network physiology has emerged [52,53]. The goal of network physiology is to horizontally integrate physiological systems where individual structures and regulation mechanisms lead to biological behavior and unique physiological functions. There is a necessity to develop innovative analytical instruments and theoretical structures to address dynamical networks observed in physiological systems, which has further underscored the need for a highly interdisciplinary ‘Team Medicine’ approach to the problem.

### 2.2. Clinical Network in Cancer Systems Biology

A Clinical Network may be likened to a complex system comprising individual physicians and physician–scientists at both academic and community practice sites who enhance cancer systems biology through biomarker discovery, translational research, and clinical trial enrollment with a focus on cross-collaborative precision medicine. Precision medicine is the tool that drives cancer systems biology, where the technologies of precision medicine are utilized in tandem with the clinical network to study the distinct biological and environmental factors of each patient toward the development of new therapeutics [54]. Precision medicine has revolutionized the field of cancer over the last two decades from identifying new cancer biomarkers, genetic alterations, and treatments to improving patient outcomes [55,56,57]. Despite all the successes, there are several shortcomings of current precision medicine that need to be addressed such as its incorporation into the clinical cancer network, more consistent serial specimen collection, and increased collaboration between the researchers and clinicians to harness the research network in real time [58,59]. Here, we introduce the clinical network as a part of cancer systems biology and build upon our approach by proposing a model for an AI-driven drug-matching algorithm.

One crucial issue that needs to be resolved for the further widespread adaptation of precision oncology is the consistent use of biomarker platforms at the community and independent oncology clinic level. The availability of biomarker testing among practicing oncologists differs based on their geographical location and practice type with reported rates varying from 0.1% to 100% in actionable biomarkers in community practices, indicating the need for further policies that ensure all cancer patients have access to precision oncology [32,60]. Limited resources at both the clinics and in the community are a few of the multiple factors that contribute to this disparity [61]. Furthermore, the utilization of multiplex biomarker tests in clinical practice varied significantly among oncologists, and since many reported mixed confidences in interpreting these results, evidence-based guidelines and deploying pathways with the combination of physician education efforts may combat this issue [62]. The implementation of large panel omics testing across the clinical network would improve biomarker discovery in cancer systems biology. A multifaceted approach is needed to encompass as many solutions as possible comprising a wide array of parameters to include infrastructure changes such as the expansion of academic centers to incorporate community clinics or geographical sites into one large oncology network, the use of clinical pathways, and the development of molecular tumor boards within those networks and at the patient level such as community engagement, education, and empowerment [61,63,64,65].

Our previous work highlighted the importance of a strong integrated clinical and research network at both academic and community practice sites [29,32,33,34,66,67]. Oncology pathways that guide physicians have been implemented across the City of Hope network, and applying such a strategy can ensure that patients are assigned appropriate therapies based on their biomarker profile both in the academic and community practice settings [66,67]. Most cancer patients start their cancer journey with a community oncologist, and the main reason they are referred to an academic site is to enroll in a clinical trial; nevertheless, cooperation and communication between sites needs to increase [68]. The complete incorporation and cross-collaboration of clinical trial systems from the lowest levels (e.g., community sites) to the highest levels (e.g., national networks) is critical in expanding access to clinical trials, which are specifically biomarker-driven [30,67]. The decentralization of clinical trials conducted in the clinical network would address disparities of care, access to care, and raise trial accrual rates that will accelerate the cancer systems biology drug discovery pipeline [69]. We have previously designed a pyramidal decision support framework that leverages this cross-collaboration through four distinct levels including a clinical pathway program, network and academic clinician consultations, disease team tumor boards, and complex oncology case discussions [33]. This would allow for a better examination of rarer cancer-type populations such as Nuclear protein of the Testis (NUT) carcinomas or narrow targets for traditionally hard-to-treat cancers such as pancreatic cancer [30,70,71,72].

Additionally, multidisciplinary cancer teams’ collaboration and sub-specialties are a vital component in the clinical network systems biology where knowledge and expertise need to be diversified beyond individual cancer specialists such as the involvement of pathologists, radiologists, and others to improve patient outcomes, particularly in complex cases and through the utilization of Precision Oncology Tumor Boards [33]. Baseline and serial sample collections need to be improved across the network. The use of technologies such as liquid biopsies and single-cell sequencing can help determine the early signs of possible recurrence of early-stage cancers, monitor treatment response, and follow the evolutionary heterogeneity diversity between cancer clones [73,74,75]. Yet, despite the prevalence and importance of biobanking protocols at institutions, many fail to capture the necessary specimens and data to accelerate its adaptation networkwide [76,77,78].

**Figure 3:**
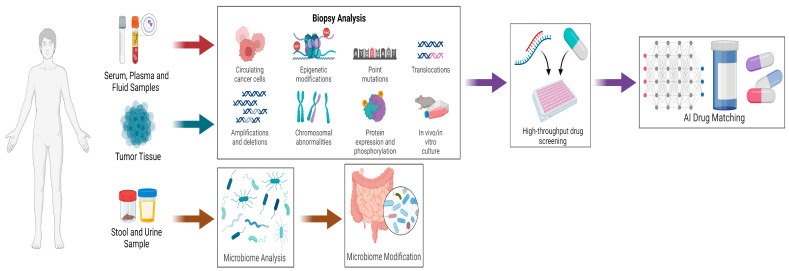
AI-driven drug-matching algorithm for future personalized medicine (created with BioRender.com, accessed on 1 May 2023).

Previously mentioned stopgaps to precision medicine and more recently personalized medicine have largely been due to the high cost of various sequencing techniques as well as the cost of drug development or repurposing, and they have been limited by a lack of high-throughput drug screening. With the advent of liquid biopsies, it is now possible to study circulating tumor cells and detect protein expression from standard blood as well as cerebrospinal fluid (CSF) in patients with leptomeningeal metastases [74,79]. Advances in microbiome analysis have resulted in the identification of temporal changes in microbiome composition as a potential marker for immunotherapy response [80]. Microbiome discoveries have resulted in novel techniques of fecal microbiota transplants and have been shown in advanced melanoma to help immunotherapy-resistant patients overcome anti-PD-1 resistance [81]. Novel biopsy analysis techniques to detect and study circulating cancer cells, epigenetic modifications, point mutations, translocations, amplifications, deletions, chromosomal abnormalities, protein expression, and phosphorylation are now more readily used for liquid and tissue samples. Alongside this, the development of rapid 3D cell cultures and tumor organoids allows for high-throughput drug screening [82,83,84]. The recent developments in artificial intelligence, specifically machine learning, can further enhance personalized drug screening and match patients quickly with appropriate therapies and discover new therapeutics or candidates for drug repurposing [82,85,86]. Taken altogether, harnessing the clinical data and specimens and the research network, we have designed and proposed a novel real-time AI-driven drug-matching algorithm that could be utilized to enhance future personalized medicine (Figure 3). Additionally, the hope is that this technology ultimately assists in drug discovery and the development of novel therapies by taking advantage of retrospective samples leading to clinical trials.

## 3. Conclusions

Cancer systems biology has been instrumental in the recent discoveries of precision medicine. Furthermore, the integration of traditional basic science and clinical cancer researchers with a multidisciplinary team of scientists from other fields has allowed for the study of cancer at multiple scales with a deeper understanding of its biology and evolution. While previously, sequencing cost remained a barrier for clinical research, novel technologies have made it possible to quantitate tumor samples beyond genomic sequencing toward understanding protein expression and phosphorylation, epigenetic, chromosomal abnormalities, and other non-genetic mechanisms in real-world clinical samples. Furthermore, adaptive therapy (also known as intermittent therapy) based on the principles of ecology and evolution may help address the issue of drug resistance, which is almost inevitable [87,88]. This has allowed for the study of cancer biology at multiple scales enhanced by the traditional experimental and computational models. However, further cross-collaboration and integration between individual academic sites, national cancer networks, and community practices is required to achieve truly personalized medicine. The implementation of these ideas powered by recent advances in artificial intelligence and machine learning would in the future allow for personalized high-throughput drug screenings that would yield faster drug discoveries and approved therapeutics.

## Data Availability

No new data were created or analyzed in this study. Data sharing is not applicable to this article.
